# The multiple mediating roles of depression and grandchild care in the relationship between social participation and cognitive function among middle-aged and elderly Chinese: an empirical study based on CHARLS 2020 data

**DOI:** 10.3389/fnagi.2025.1549615

**Published:** 2025-03-27

**Authors:** Sumei Zhou, Tianfang Deng, Shirong Shao, Zhi Zeng

**Affiliations:** ^1^Department of Neurosurgery, Deyang People’s Hospital, Deyang, Sichuan, China; ^2^Department of Gastroenterology, Deyang People’s Hospital, Deyang, Sichuan, China

**Keywords:** cognitive function, social participation, middle-aged and elderly, grandchild care, depression, CHARLS, mediation effect study

## Abstract

**Background:**

With global and Chinese populations aging rapidly, maintaining cognitive function among middle-aged and elderly individuals has become a critical health priority. Understanding the factors influencing cognitive health is crucial for developing effective health policies and interventions.

**Objectives:**

This study investigates the impact of social participation on cognitive function among middle-aged and elderly individuals, examining the mediating effects of depression and grandchild care. Given the potential variation in these effects across different demographic and socioeconomic groups, this study also explores subgroup differences to provide targeted policy recommendations.

**Methods:**

Data from the 2020 China Health and Retirement Longitudinal Study (CHARLS) were used to analyze the relationship between social participation and cognitive function. Ordinary Least Squares (OLS) and stepwise regression models were employed, with robustness checks conducted using 2SLS regression.

**Results:**

The study included 17,962 participants aged 45 and above. Baseline regression results indicate that social participation significantly enhances cognitive function (β = 0.417, *p* = 0.001) after adjusting for confounding factors. Subgroup analysis revealed that the cognitive benefits of social participation were more pronounced among individuals residing in the western region, those aged 60 and above, high-income groups, and rural populations. Mediation analysis showed that depression played a more substantial mediating role (β = –0.109, *p* = 0.001), while grandchild care exhibited a statistically significant but relatively small mediation effect (β = 0.004, *p* = 0.001).

**Conclusion:**

Social participation not only directly improves cognitive function but also indirectly enhances it by reducing depression and increasing grandchild caregiving engagement among middle-aged and elderly individuals. However, while the mediating effect of grandchild care is statistically significant, its effect size remains relatively small, suggesting that its overall contribution to cognitive function should be interpreted with caution. In contrast, depression demonstrates a more substantial mediating effect, highlighting the critical role of mental health in cognitive aging. Given these findings, policy should prioritize interventions that mitigate depression as a primary pathway for enhancing cognitive function in aging populations. Expanding social participation opportunities should be a key strategy, particularly in the central and eastern regions, among individuals under 60 years old, those with lower income levels, and urban residents, to ensure equitable access to cognitive health benefits. Additionally, while grandchild caregiving may serve as a meaningful form of social engagement, its effects on cognitive function appear to be complex and context-dependent. Future research should explore the long-term impact of caregiving intensity and emotional burden on cognitive health to better inform aging policies and intergenerational support programs.

## 1 Introduction

Cognitive function refers to the brain’s ability to process, store, and retrieve information, serving as a crucial indicator of individual physical and mental health as well as quality of life (Koczwara et al., 2012). With the rapid global aging trend, cognitive decline has become a pressing public health concern, particularly among middle-aged and elderly individuals. Neurodegenerative diseases such as dementia and Alzheimer’s disease are on the rise, posing significant challenges to healthcare systems worldwide (Fang et al., 2015). By 2050, it is estimated that over 130 million people globally will suffer from dementia (Di Tella et al., 2021; Fabbri et al., 2018). In China, the annual increase in dementia cases reaches 300,000, constituting a quarter of the global total and ranking first worldwide (Hendriks et al., 2021). The increasing prevalence of dementia not only affects individuals and families but also places a substantial economic burden on healthcare and social welfare systems (Lv et al., 2019; Wang, 2023). Projections indicate that by 2030, the annual treatment and care costs for dementia patients in China will reach $11.42 billion USD (Xu et al., 2017). Given the urgent need to address cognitive decline in aging populations, identifying protective factors and intervention strategies has become a critical research priority.

As the country with the largest elderly population globally, China is facing increasingly severe population aging and health challenges (Wang M. et al., 2022). To address these challenges, the Chinese government has implemented a series of aging policies to actively promote social participation among the elderly, aiming for a higher level of “Productive Aging” (Fang et al., 2020). The World Health Organization (WHO) advocates for “active aging,” emphasizing the three pillars of health, participation, and security to improve the quality of life of the elderly and mitigate the negative impacts of population aging (Bourassa et al., 2017). Within this framework, social participation is seen as an important health promotion measure, and its impact on cognitive function in the elderly has received widespread attention (Chen et al., 2024; Fu et al., 2018; Gu et al., 2024).

### 1.1 Social participation and cognitive function

Social participation, defined as engagement in community activities, volunteer work, and social interactions, has been recognized as a key determinant of cognitive health among older adults (Barragán et al., 2021; Chopik, 2016; Mao et al., 2020; Sheng et al., 2024). According to the Use and Disuse Theory (Wang J. et al., 2024), cognitive abilities follow the principle of “use it or lose it,” meaning that reduced social engagement accelerates cognitive decline, whereas active participation in mentally and physically stimulating activities helps maintain cognitive function. The Cognitive Reserve Hypothesis further suggests that individuals who frequently engage in social interactions build greater cognitive resilience, enhancing neural efficiency and reducing the risk of dementia (Kremen et al., 2022). Additionally, Social Identity Theory posits that social participation fosters a sense of belonging, enhances self-esteem, and promotes emotional wellbeing, all of which contribute to better cognitive performance (Lam et al., 2020).

Despite growing evidence highlighting the cognitive benefits of social participation, research on its underlying mechanisms remains insufficiently explored (Brown et al., 2012; Su et al., 2018; Wilson et al., 2007). Prior studies suggest that the relationship between social engagement and cognitive function is not direct but rather mediated by psychological and social factors (George et al., 2020; Shah et al., 2016). Understanding these mediating pathways is essential for developing targeted interventions to promote cognitive health in aging populations.

### 1.2 The mediating role of depression

Depression is a well-established risk factor for cognitive decline, and it may serve as a key mediator in the relationship between social participation and cognitive function (Hou et al., 2023; Kang et al., 2018). Engaging in social activities can provide emotional support, reduce loneliness, and increase feelings of self-worth, all of which help prevent or alleviate depression (Gao et al., 2023; Li et al., 2022). Conversely, social isolation has been linked to higher levels of depression, which in turn negatively affects cognitive function by impairing memory, attention, and executive functioning (Wang S. et al., 2024). Thus, depression may act as a mediating mechanism through which social participation bolsters cognitive function.

### 1.3 The mediating role of grandchild care

Another important factor is grandchild care, which represents a unique form of social participation, particularly in cultures that emphasize intergenerational support (Coall and Hertwig, 2010). In China, many middle-aged and elderly individuals assume caregiving responsibilities for their grandchildren, which can provide both cognitive stimulation and emotional fulfillment (Wang S. et al., 2022).

Studies suggest that moderate levels of grandchild care may enhance cognitive function by increasing daily activity levels, providing emotional engagement, and maintaining social interactions (Rafael et al., 2021). However, excessive caregiving responsibilities may lead to negative effects, as proposed by Role Strain Theory (Hughes et al., 2007). Intensive childcare duties can cause physical and psychological stress, reduce opportunities for personal leisure and exercise, and contribute to increased depressive symptoms (Zeng et al., 2021). Additionally, conflicts with younger parents over parenting styles and intergenerational differences in caregiving expectations may further contribute to psychological distress (Samuel et al., 2017). These negative outcomes can, in turn, impair cognitive function, creating a bidirectional relationship between grandchild care and cognitive health. Thus, grandchild care may serve as a mediating factor in the relationship between social participation and cognitive function, with its effects contingent on the balance between the benefits of engagement and the burdens of caregiving.

### 1.4 Research hypotheses

**FIGURE 1:**
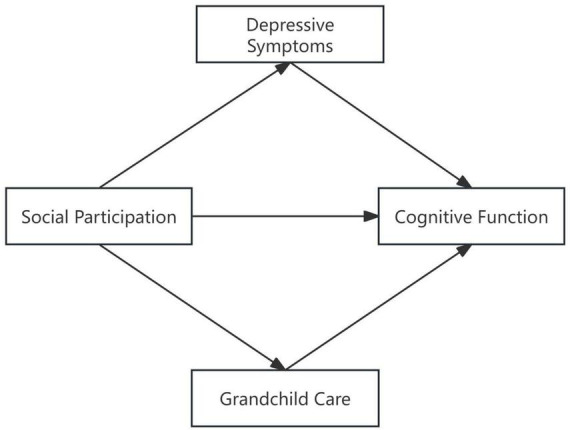
The proposed mediated model.

Based on these theoretical perspectives, this study seeks to clarify the complex relationships between social participation, cognitive function, depression, and grandchild care. The research aims to test the following hypotheses (Figure 1).

Hypothesis 1 (H1): Social participation is positively associated with cognitive function among middle-aged and elderly individuals.

Hypothesis 2 (H2): Depression mediates the relationship between social participation and cognitive function among middle-aged and elderly individuals.

Hypothesis 3 (H3): Grandchild care serves as a mediator in the social participation-cognitive function relationship among middle-aged and elderly individuals.

To empirically test these hypotheses, we will utilize data from the 2020 China Health and Retirement Longitudinal Study (CHARLS), a nationally representative dataset. Through constructing a theoretical model and employing Ordinary Least Squares (OLS) and stepwise regression models for empirical testing, we aim to investigate the impact of social participation on cognitive function among middle-aged and elderly individuals. Additionally, we will analyze the mediating roles of depression and grandchild care in this relationship. This research endeavors to provide insights for further implementation of strategies promoting social participation in aging populations and advancing healthy aging initiatives.

## 2 Research design

### 2.1 Data source and processing

The data used in this study originates from the 2020 (wave 5) of the China Health and Retirement Longitudinal Study (CHARLS) database. CHARLS is a nationwide longitudinal survey that covers 28 provinces, municipalities, and autonomous regions in China, encompassing approximately 17,000 households and 28,000 respondents (Zhao et al., 2014). Utilizing a multistage stratified probability sampling method, the survey collected samples from middle-aged and elderly populations across different regions, urban and rural areas, and socioeconomic backgrounds. Its primary objective is to gain a deeper understanding of the social, economic, and health statuses of individuals aged 45 and above, making it highly representative of the national population. All data is accessible on the Peking University Open Research Data Platform.

**FIGURE 2:**
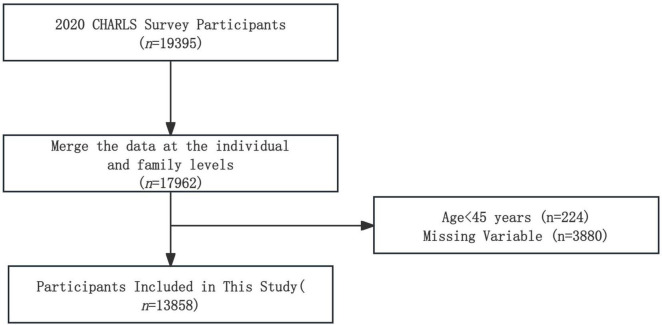
Sample selection flowchart.

In the data processing phase, this study merged individual and household-level data, initially obtaining 17,962 samples. Subsequently, samples below the age of 45 years were excluded, resulting in 17,738 remaining samples. Further exclusions were made for samples with missing variable values, as well as missing core explanatory variables, resulting in a final effective sample size of 13,858. For detailed sample selection procedures, as presented in Figure 2.

However, the 2020 CHARLS survey did not include specific items related to grandchild care. To address this limitation, we applied a retrospective data processing approach by incorporating grandchild care data from the same respondents in the 2018 CHARLS (Wave 4) and matching individuals using their unique ID numbers. A unique individual ID matching method was employed to track middle-aged and elderly respondents who reported providing grandchild care in 2018. Given that grandchild caregiving is a long-term, structured family responsibility in Chinese society, it is reasonable to assume that many of these individuals continued providing care in 2020. By leveraging this approach, we approximate grandchild care status in 2020 while maintaining a large and representative sample size.

### 2.2 Variable selection

#### 2.2.1 Dependent variable

This study selects cognitive function as the dependent variable. In the CHARLS 2020 survey, cognitive function was assessed in two dimensions: episodic memory and mental status (Xu et al., 2024). Episodic memory was evaluated using immediate word recall (0–10 points) and delayed word recall (0–10 points). Mental status assessment included three aspects: time orientation, calculation ability, and drawing ability. It evaluated the respondent’s ability to state the current date (month, day, year), day of the week, season, perform serial subtraction of seven from 100 (five times), and redraw a previously shown figure. Scores range from 0 to 11, with higher scores indicating superior mental status.

The total cognitive function score is computed as the sum of episodic memory and mental status scores, ranging from 0 to 31 points, with higher scores indicating better cognitive function.

#### 2.2.2 Explanatory variable

The central explanatory variable in this study is social participation. The participants’ recent engagement in social activities over the past month was assessed, including the following: (1) visiting friends or relatives; (2) playing mahjong, chess, cards, or attending community activities; (3) providing help to non-cohabiting relatives, friends, or neighbors; (4) participating in dancing, fitness, or qigong; (5) involvement in club activities; (6) volunteering, charity work, or caregiving for non-cohabiting patients or disabled individuals; and (7) attending school or training courses. The level of social participation is determined by summing the number of social activities in which the participants engaged, with a total score ranging from 0 to 7 points.

#### 2.2.3 Mediating variable

This study considers depression and grandchild care as mediating variables. In the CHARLS 2020 survey:(1) Depression was assessed using the Center for Epidemiologic Studies Depression Scale (CES-D), which evaluated participants’ depressive symptoms over the past week. The scale comprises 10 self-reported items, comprising two positively worded items and eight negatively worded items. Responses range from “rarely or none of the time” to “most or all of the time,” scored from 0 to 3. Positive items are reverse-scored. Total scores range from 0 to 30, where scores ≥ 10 indicate depressive symptoms and scores < 10 indicate the absence of significant symptoms. Higher scores indicate more severe depression. (2) The concept of “grandchild care” was operationalized based on responses to the following question: “In the past year, how much time per week did you and your spouse spend taking care of children?” The aforementioned time spent represents the variable of grandchild care (Liao et al., 2021).

#### 2.2.4 Control variables

Based on previous literature, the control variables included in this study are categorized into individual and family levels (Chen et al., 2024; Ma et al., 2020). The variables at the individual level encompass age, gender, level of education, marital status, health status, smoking, drinking, and chronic conditions. On the other hand, the control variables at the family level include family size, family income, family consumption, and number of children. Detailed information on variable selection is provided in Table 1.

**TABLE 1:** Categories, names, and descriptions of variables.

Variable type	Variable name	Variable symbol	Variable description
Dependent variable	Cognitive function	mmse	Continuous variable, 0–31 points
Independent variable	Social participation	Socialnum	Continuous variable, 0–7 points
Mediating variable	Grandchild care	Caretime	Continuous variable
	Depression	cesd	Continuous variable, CES-D scale, higher scores indicate higher severity
**Individual Level**			
Control variables	Age	Age	Continuous variable, actual age of respondents
	Gender	Gender	Male = 1, female = 0
	Level of education	Eduyear	No formal education = 0 years, primary school = 6 years, junior high school = 9 years, high school = 12 years, technical secondary school = 13 years, junior college = 15 years, bachelor’s degree = 16 years, master’s degree = 19 years, doctoral degree = 22 years
	Marital status	Marry	Married/cohabiting = 1, Separated/divorced/widowed/single = 0
	Health status	Health	Very poor = 1, poor = 2, fair = 3, good = 4, excellent = 5
	Smoking	Smoke	Yes = 1, no = 0
	Drinking	Drink	Yes = 1, no = 0
	Chronic conditions	Diag	Yes = 1, no = 0
**Family level**			
Control variables	Family size	Family size	Continuous variable, total family size
	Family income	Income	Continuous variable, total household income plus 1, logarithm
	Family consumption	PCE	Continuous variable, household consumption plus 1, logarithm
	Number of children	Child	Continuous variable, total number of children in the family

The 2020 survey did not include items related to grandchild care. This study retrospectively processed the data for this variable by substituting data from the same individual-related questions in the 2018 sample into the 2020 data set.

### 2.3 Model construction

#### 2.3.1 Baseline regression: ordinary least squares (OLS) model

This study employs Ordinary Least Squares (OLS) to estimate the impact of social participation on cognitive function among middle-aged and elderly individuals. The specific model setup is as follows:


(1) $$m\,m\,s\,e_{i}=\beta_{0}+\beta_{1}\,s\,o\,c\,i\,a\,l\,n\,u\,m_{i}+\beta_{n}\,c\,o\,n\,t\,r\,o\,l_{i}+\varepsilon_{i}$$

#### 2.3.2 Examination of mediating variables: stepwise regression model

Stepwise regression method is used to verify the multiple mediating effects of grandchild care and depression on social participation and cognitive function among middle-aged and elderly individuals. The specific model setup is as follows:


(2) $$c\,a\,r\,e\,t\,i\,m\,e_{i}=\beta_{0}+\beta_{1}\,s\,o\,c\,i\,a\,l\,n\,u\,m_{i}+\beta_{n}\,c\,o\,n\,t\,r\,o\,l_{i}+\varepsilon_{i}$$

(3) $$m\,m\,s\,e_{i}=\beta_{0}+\beta_{1}\,s\,o\,c\,i\,a\,l\,n\,u\,m_{i}+\beta_{2}\,c\,a\,r\,e\,t\,i\,m\,e_{i}+\beta_{n}\,c\,o\,n\,t\,r\,o\,l_{i}+\varepsilon_{i}$$

(4) $$c\,e\,s\,d_{i}=\beta_{0}+\beta_{1}\,s\,o\,c\,i\,a\,l\,n\,u\,m_{i}+\beta_{n}\,c\,o\,n\,t\,r\,o\,l_{i}+\varepsilon_{i}$$

(5) $$m\,m\,s\,e_{i}=\beta_{0}+\beta_{1}\,s\,o\,c\,i\,a\,l\,n\,u\,m_{i}+\beta_{2}\,c\,e\,s\,d_{i}+\beta_{n}\,c\,o\,n\,t\,r\,o\,l_{i}+\varepsilon_{i}$$

Note: *mmse*_*i*_ represents an individual’s cognitive function, *socialnum*_*i*_ represents an individual’s social participation situation, *caretime*_*i*_ represents an individual’s grandchild care situation, *cesd*_*i*_ represents the level of depression in an individual, *i* denotes different individuals, *control*_*i*_ represents controlling variables, ε_*i*_ represents random error, β_0_,β_1_,β_2_,β_*n*_ represents estimating coefficients.

### 2.4 Ethical approval

This study has been approved by the Peking University Biomedical Ethics Committee (IRB00001052-11015), and informed consent has been obtained from all participants.

### 2.5 Descriptive statistics

Table 2 presents descriptive statistics for each variable. (i) Dependent and Independent Variables: The mean cognitive function score among the middle-aged and elderly individuals is 15.743, indicating a moderate level. The core independent variable, social participation, exhibits a mean of 0.786 and a standard deviation of 1.004. Approximately 49.15% of the middle-aged and elderly participated in social activities in the past month, indicating an overall positive attitude toward social participation among the sample, albeit with limited types of social activities. (ii) Sample Personal-Level Variables: The mean age of the sample is 61.104 years, with a gender ratio of 0.473. The mean number of years of education is 4.85, while 86% of respondents are married/cohabiting. The average self-reported health status is 3.046, with 81.3% reporting at least one chronic condition. These figures suggest a relatively low educational level and widespread health concerns among the sample. (iii) Sample Family-Level Variables: The average household size is 2.515 individuals, with 2.504 children per household. Mean household income and expenditure are 9.226 and 10.354, respectively, indicating economic pressures that may impact cognitive function.

**TABLE 2:** Descriptive statistics.

Variable	Mean	SD	Min	Max	Obs.
Cognitive function	15.743	6.206	0	31	13,858
Social participation	0.786	1.004	0	7	13,858
Age	61.104	9.209	45	108	13,858
Gender	0.473	0.499	0	1	13,858
Level of education	4.85	4.636	0	19	13,858
Marital status	0.86	0.347	0	1	13,858
Health status	3.046	1.028	1	5	13,858
Smoking	0.423	0.494	0	1	13,858
Drinking	0.365	0.481	0	1	13,858
Chronic conditions	0.813	0.390	0	1	13,858
Family size	2.515	1.232	1	12	13,858
Family income	9.226	2.661	0	14.04	13,858
Family consumption	10.354	1.000	0	13.956	13,858
Number of children	2.504	1.247	0	10	13,858

## 3 Empirical results and analysis

### 3.1 Multicollinearity test

To verify multicollinearity among explanatory and control variables, a variance inflation factor (VIF) test was conducted. All VIF values remained below 10, indicating no multicollinearity concerns. Thus, variable selection in this study is statistically appropriate (Table 3).

**TABLE 3:** Results of multicollinearity test for each variable.

Variable	Variance inflation factor (VFI)	1/VFI
Gender	2.56	0.389893
Smoking	2.24	0.445568
Age	1.54	0.648489
Number of children	1.34	0.746652
Family consumption	1.29	0.773346
Drinking	1.29	0.776748
Level of education	1.28	0.780526
Marital status	1.21	0.823382
Family size	1.18	0.848649
Family income	1.17	0.853606
Health status	1.14	0.877930
Chronic conditions	1.13	0.886860
Social participation	1.08	0.928239
Mean variance inflation factor	1.42	

### 3.2 Baseline model regression analysis

Table 4 presents the baseline regression results on the impact of social participation on cognitive function among middle-aged and elderly individuals (Equation 1).

**TABLE 4:** Baseline regression result.

Variable	Cognitive function		
	**Model 1**	**Model 2**	**Model 3**
Social participation	1.250***	0.463***	0.417***
	(24.315)	(10.574)	(9.533)
Age	–	–0.130***	–0.111***
		(–25.360)	(–19.441)
Gender	–	0.144	0.230*
		(1.054)	(1.694)
Level of education	–	0.611***	0.578***
		(60.354)	(55.976)
Marital status	–	1.091***	0.901***
		(8.378)	(6.709)
Health status	–	0.236***	0.190***
		(5.373)	(4.323)
Smoking	–	–0.083	–0.121
		(–0.641)	(–0.940)
Drinking	–	0.404***	0.325***
		(4.034)	(3.263)
Chronic conditions	–	0.411***	0.383***
		(3.537)	(3.322)
Family size	–	–	–0.093**
			(–2.483)
Family income	–	–	0.157***
			(9.128)
Family consumption	–	–	0.272***
			(5.647)
Number of children	–	–	–0.242***
			(–6.171)
Constant	14.760***	18.207***	14.126***
	(225.069)	(44.260)	(21.914)
*N*	13,858	13,858	13,858
*R* ^2^	0.041	0.347	0.356

*, **, and *** denote significance levels of 10%, 5%, and 1%, respectively.

Model 1 shows that without control variables, social participation significantly enhances cognitive function, with an increase of one type of social activity linked to a 1.250-point rise in cognitive function scores. Model 2 and 3, which introduce individual and family-level control variables, confirm that the positive effect remains highly significant at the 1% level. This finding underscores the robust association between social participation and cognitive function, suggesting that engagement in social activities meaningfully contributes to cognitive wellbeing in aging populations.

Individual-Level Influences on Cognitive Function: Age exhibits a significant negative effect on cognitive function. As individuals age, there are physiological changes in brain structure and function occur, including neuronal loss, slowed synaptic transmission, and diminished neuroprotective mechanisms. These changes contribute to declines in memory, slower thinking, and reduced cognitive processing speed, thereby adversely affecting cognitive function in this demographic (Shu et al., 2021). Higher education levels are positively associated with cognitive function. Higher levels of educational attainment provide individuals with a greater range of cognitive stimulation and learning experiences, which in turn facilitate enhanced information processing abilities and cognitive flexibility, thereby slowing the rate of cognitive decline (Wang Y. et al., 2024). Married/cohabiting middle - aged and elderly people tend to have better cognitive function, perhaps due to stable marital relationships, more social support, and better mental wellbeing (Van Bogart et al., 2024). Certain chronic conditions such as diabetes and cardiovascular diseases may also have a positive impact on cognitive function, potentially influencing brain health through various pathways (Shu et al., 2024; Tarraf et al., 2020). Furthermore, there is evidence to suggest that moderate long-term alcohol consumption is associated with enhanced cognitive function. This effect may be attributed to the elevation of brain-derived neurotrophic factor levels by moderate alcohol intake, along with the positive effects of certain alcohol compounds on neuroprotection and cardiovascular health (Zhang et al., 2020).

Family-Level Influences on Cognitive Function: Larger household size and more children negatively correlate with cognitive function. Resource dispersion, increased family responsibilities, and psychological strain may contribute to cognitive decline (Du et al., 2023; Yang et al., 2022). Higher household income and expenditure positively impact cognitive function. Better financial security enhances access to healthcare, nutritious diets, and cognitively stimulating activities, thereby promoting cognitive resilience (Kim and Lee, 2019; Liu et al., 2022; Shi et al., 2023).

### 3.3 Heterogeneity analysis

The promoting effect of social participation on cognitive function among middle-aged and elderly individuals may exhibit heterogeneity across sample characteristics. This study employed grouped regressions based on different geographical regions, age groups, household incomes, and urban-rural divides to further examine the differential impact of social participation on cognitive function among different groups. The results are presented in Table 5.

**TABLE 5:** Heterogeneity analysis results.

Variable	Cognitive function									
	**Eastern region**	**Central region**	**Western region**	**Middle age**	**Elderly**	**Low income**	**Middle income**	**High income**	**City**	**Village**
Social participation	0.451***	0.321***	0.531***	0.341***	0.600***	0.037***	0.014*	0.353***	0.343***	0.452***
	(5.631)	(4.730)	(6.459)	(6.168)	(8.668)	(3.010)	(1.659)	(5.794)	(5.402)	(7.645)
Control variables	Yes	Yes	Yes	Yes	Yes	Yes	Yes	Yes	Yes	Yes
Constant	16.075***	13.854***	12.419***	6.780***	6.729***	15.208***	15.963***	17.252***	13.317***	16.146***
	(14.496)	(13.222)	(10.293)	(8.761)	(9.676)	(13.252)	(12.698)	(14.684)	(12.947)	(19.280)
*N*	4,772	5,224	3,862	6,318	7,540	4,628	4,667	4,563	4,928	8,930
*R* ^2^	0.327	0.361	0.378	0.291	0.297	0.282	0.276	0.333	0.350	0.337

* and *** denote significance levels of 10% and 1%, respectively. The control variables are consistent with the baseline regression and thus omitted here. The same below.

Regional Heterogeneity Analysis: Social participation significantly enhances cognitive function across all regions, with the strongest effect observed in western China. This may be attributed to the region’s lower socioeconomic development and slower pace of life, which provide more opportunities for social engagement and emotional support. Furthermore, strong cultural traditions emphasizing community and family cohesion may further facilitate participation in social activities, thereby amplifying its cognitive benefits (Everly et al., 2023; Kelly et al., 2017).

Age Heterogeneity Analysis: Dividing the sample into middle-aged (45–59 years) and elderly (60^+^ years) groups reveals that social participation benefits both, with a more pronounced effect among the elderly. Cognitive decline is more prevalent with aging, and social participation provides essential cognitive stimulation, helping mitigate this decline. Additionally, post-retirement engagement in social activities helps fill social gaps, fostering continued cognitive engagement (Posis et al., 2024; Richards et al., 2019).

Family Income Heterogeneity Analysis: Social participation has a stronger effect on cognitive function among higher-income individuals. Wealthier households have better access to diverse social networks and recreational activities, offering more opportunities for cognitive stimulation and emotional support (Smith et al., 2022; Zhu et al., 2023).

Urban-Rural Heterogeneity Analysis: While social participation benefits cognitive function in both urban and rural populations, the effect is stronger in rural areas. This may be due to limited access to educational resources, healthcare, and organized social activities in rural areas, making informal social interactions particularly vital for cognitive stimulation and emotional support. Moreover, rural life is relatively simpler, with fewer daily obligations and stronger social ties, allowing for more frequent interpersonal interactions. The prevalence of close-knit communities and familiar social circles increases opportunities for informal gatherings, fostering higher social participation, stronger emotional connections, and greater cognitive benefits (Harling et al., 2020).

### 3.4 Robustness and endogeneity tests

#### 3.4.1 Robustness test

To confirm the robustness of the regression results, several alternative specifications were employed (Table 6):

**TABLE 6:** Robustness test results.

Variable	Cognitive function		
	**Model 1**	**Model 2**	**Model 3**
Whether to participate in social activities	0.829*** (9.604)	–	–
Social participation	–	0.288***	0.443***
		(9.318)	(8.889)
Control variables	Yes	Yes	Yes
Constant	14.043***	10.122***	12.143***
	(21.785)	(22.235)	(14.712)
*N*	13,858	13,858	11,013
*R* ^2^	0.356	0.245	0.330

*** denote significance levels of 1%.

Model 1 - Substituting explanatory variables: Considering whether participation in social activities is also an effective variable for measuring the level of social activity participation, we elected to include participation in social activities as an explanatory variable for further validation. The promotional effect of social activity participation on cognitive abilities was confirmed through significance testing, demonstrating robust results.

Model 2 - Substituting response variables: Memory capacity was chosen as the response variable to further validate cognitive ability measurement. The regression coefficients remained significant, ensuring robustness of the study’s conclusions.

Model 3 - Sample selection: Samples with excessively low or high household incomes might influence the study. After excluding the top 10% and bottom 10% of household income samples, the coefficient for social activity participation passed significance testing, with all coefficients positive, indicating robust and reliable results.

#### 3.4.2 Endogeneity test

Considering the potential endogeneity issues affecting the effect of social activity participation on cognitive abilities in middle-aged and elderly individuals, such issues could arise from measurement errors, omitted variables, and reverse causality. To address endogeneity, this study employed the instrumental variable (IV) approach, using the average level of social participation in other samples from the same city as instruments for social activity participation.

Wald test results (*p* = 0.000) confirm the presence of endogeneity. The first-stage IV regression (Table 7) shows that the instrument is strongly correlated with social participation (1% significance level). Cragg-Donald Wald F statistic results confirm that weak instrument bias is not an issue.

**TABLE 7:** Endogeneity test results.

Variable	The first-stage regression in the IV method	The second-stage regression in the IV method
	**Social participation**	**Cognitive function**
Instrumental variable	0.645*** (16.425)	–
Social participation	–	2.148***
		(6.448)
Control variables	Yes	Yes
Constant	–0.308**	14.120***
	(–2.454)	(20.040)
*N*	13,858	13,858
*R* ^2^	0.090	0.283
First-stage F statistic (*p*-value)	231.31 (0.000)	–
Wald test (*p*-value)	47.74 (0.000)	–

**, and *** denote significance levels of 5%, and 1%, respectively.

## 4 Further discussion: mediating analysis

Table 8 presents the results of the mediation effect regression for depression level and grandchild care. Model 1 estimates the effect of social participation on grandchild care (Equation 2). Model 2 evaluates the combined effects of social participation and grandchild care on cognitive function (Equation 3). Model 3 examines the impact of social participation on depression (Equation 4). Model 4 assesses the joint effects of social participation and depression on cognitive function (Equation 5).

**TABLE 8:** Mediation effect regression results.

Variable	Grandchildcare	Cognitive function	Depression	Cognitive function
	**Model 1**	**Model 2**	**Model 3**	**Model 4**
Social participation	1.409***	0.411***	–0.189***	0.396***
	(4.833)	(9.399)	(–3.799)	(9.126)
Grandchild care	–	0.004***	–	–
		(3.149)	–	–
Depression	–	–	–	–0.109***
				(–14.751)
Control variables	Yes	Yes	Yes	Yes
Constant	16.537***	14.059***	20.331***	16.347***
	(3.851)	(21.806)	(27.696)	(24.877)
*N*	13,858	13,858	13,858	13,858
*R* ^2^	0.087	0.357	0.218	0.366
Sobel test	2.639 (0.008)		3.679 (0.000)	

*** denote significance levels of 1%.

Model 1 indicates that social participation significantly increases grandchild caregiving engagement. Model 2 further reveals that both grandchild care and social participation positively impact cognitive function, suggesting that elderly individuals who actively engage in social activities are more likely to take on caregiving roles, which, in turn, may provide cognitive stimulation through intergenerational interactions.

However, it is important to note that although the mediation effect of grandchild care is statistically significant (*p* = 0.001), its effect size (β = 0.004) is relatively small. This suggests that, while caregiving offers cognitive engagement opportunities, its overall contribution as a mediator is limited. These findings align with Role Strain Theory, which posits that while caregiving can be cognitively stimulating, it may also impose stress and time burdens that counteract its benefits. Thus, caution should be exercised in interpreting its role as a key mediating factor.

Combining these with the results of the Sobel test, it can be inferred that grandchild care plays a partial mediating role in the relationship between social participation and cognitive function. That is, social participation not only has a direct positive impact on cognitive abilities but also indirectly enhances them through caregiving engagement. Despite its modest effect size, grandchild care remains an important form of social participation, fostering emotional fulfillment, intergenerational bonding, and cognitive engagement. Future research should explore how factors such as caregiving frequency, emotional strain, and the availability of family support influence the cognitive outcomes of caregiving. In contrast, depression appears to be a more substantial mediator in this relationship.

Regarding depression level, Model 3 illustrates that social participation exerts a significant inhibitory effect, exhibiting a negative correlation. Model 4 shows that social participation positively affects cognitive abilities, whereas depression level has a significant negative impact on them. This suggests that elderly individuals who participate in social activities tend to experience lower levels of depression. Social participation provides emotional support, fosters a sense of belonging, and reduces loneliness, all of which contribute to improved mental health and, subsequently, better cognitive outcomes.

Compared to grandchild care, depression demonstrates a stronger mediating effect (*p* = 0.001, β = –0.109), indicating that improving mental wellbeing may be a more critical pathway through which social participation enhances cognitive function. This aligns with the Cognitive Reserve Hypothesis, which suggests that reducing psychological distress can strengthen cognitive resilience by preserving neural function and delaying cognitive decline.

**FIGURE 3:**
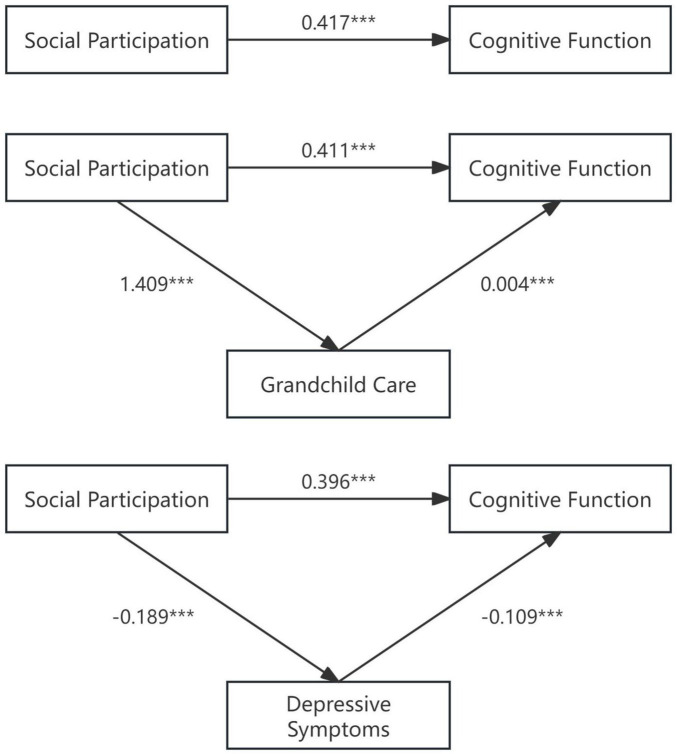
Mediation path diagram. *** denote significance levels of 1%.

The Sobel test results provide evidence that social participation contributes to cognitive function improvements in the elderly through multiple mediation pathways. Among them, alleviating depression emerges as a more substantial mechanism compared to grandchild care. The detailed path coefficients and mediation model are illustrated in Figure 3.

## 5 Conclusion and recommendations

### 5.1 Conclusion

The present study, through a combination of theoretical analysis and empirical testing, has reached the following conclusions: (i) Social participation has been demonstrated to exert a considerable positive influence on the cognitive function of middle-aged and elderly individuals, notably enhancing their cognitive abilities. Specifically, after controlling for various confounding variables, each additional type of social participation activity increases the cognitive function score by 0.417 points in middle-aged and elderly individuals. (ii) The impact of social participation on cognitive function exhibits heterogeneity. Overall, social participation positively affects middle-aged and elderly groups across different geographical regions, age cohorts, family income levels, and urban-rural living conditions. It is noteworthy that individuals residing in western regions, those aged 60 and above, higher-income groups, and rural elderly populations experience particularly pronounced benefits. (iii) Social participation indirectly affects cognitive function through two primary pathways: depression and grandchild caregiving. Increased social participation helps alleviate depressive symptoms, which in turn has a significant positive effect on cognitive function. This aligns with the Cognitive Reserve Hypothesis (Kremen et al., 2022), which suggests that engaging in social activities strengthens cognitive resilience by reducing psychological distress and promoting neuroprotective mechanisms. Compared to grandchild care, depression serves as a more substantial mediator, indicating that improvements in mental wellbeing may be a key mechanism linking social engagement to cognitive health. (iii) Although grandchild caregiving provides opportunities for cognitive engagement, its mediating effect is relatively modest. While social participation enhances the frequency of caregiving, its impact on cognitive function is limited in magnitude. According to Role Strain Theory (Hughes et al., 2007), caregiving responsibilities may impose psychological stress and time constraints, potentially counteracting the cognitive benefits of social participation. These findings highlight the dual role of caregiving, warranting further investigation into how factors such as caregiving intensity, emotional burden, and social support shape its impact on cognitive aging.

Overall, the results of this research confirm that social participation plays a critical role in maintaining cognitive function among middle-aged and elderly individuals. Nevertheless, the magnitude of its advantages varies among different population subgroups. “Vulnerable” groups, such as those residing in the central and eastern regions, middle-aged people, individuals with low-to-moderate incomes, and the urban elderly population, may encounter obstacles when attempting to access social engagement opportunities.

### 5.2 Recommendations

#### 5.2.1 Enhancing social participation opportunities

Middle-aged and elderly individuals should be encouraged and supported in actively engaging in social activities to enhance both the diversity and frequency of their participation. It is recommended that governments, communities, and families collaborate to provide a variety of social engagement opportunities, including interest groups, volunteer services, and cultural exchanges, in order to meet the diverse social needs of older adults.

#### 5.2.2 Targeted social participation strategies

Social participation strategies should be tailored to regional and demographic differences, considering variations in age, income level, and urban-rural residency. Particular attention should be given to individuals in central and eastern regions, those under 60 years old, low-income groups, and urban elderly populations, as these groups may face greater barriers to participation. Targeted interventions, such as community-based social programs and financial incentives, should be prioritized to ensure equitable access to social engagement opportunities.

#### 5.2.3 Integrating mental health into cognitive health interventions

Beyond promoting social participation, addressing mental health should be central to cognitive health interventions. Psychological counseling and social support services have been shown to alleviate depressive symptoms, thereby enhancing cognitive resilience. Given depression’s stronger mediating effect compared to grandchild caregiving, reducing social isolation and improving psychological wellbeing should be prioritized as key strategies for cognitive health preservation. Expanding community-based mental health programs and fostering multidisciplinary collaborations among policymakers and healthcare providers will ensure comprehensive and sustainable cognitive health interventions.

#### 5.2.4 Reevaluating the role of grandchild care in cognitive aging

While grandchild caregiving is a form of social engagement, its cognitive benefits are context-dependent. Moderate caregiving may provide cognitive stimulation, but excessive responsibilities can induce stress, potentially offsetting these benefits. Thus, policies promoting caregiving should not assume universal cognitive advantages but rather consider the complexities of caregiving-related stress and time constraints. Future research should adopt longitudinal approaches to examine how grandchild caregiving intensity, emotional burden, and duration affect cognitive health. A more nuanced, evidence-based framework is needed to balance the cognitive benefits and potential psychological costs of grandchild caregiving, ensuring that intergenerational support policies promote both family wellbeing and cognitive longevity.

## 6 Limitations and future directions

This study has several limitations. First, our analysis relies on self-reported measures, which may introduce reporting bias. Future research should incorporate objective cognitive assessments to validate our findings.

Second, despite employing robustness checks and instrumental variable analysis, the cross-sectional design limits causal inference. Future research should incorporate longitudinal studies and experimental interventions to establish a more definitive causal relationship.

Furthermore, although the mediation effect of grandchild care was statistically significant, its effect size was minimal. This may stem from the complexity of intergenerational caregiving and its varying influence on cognitive function. Additionally, the temporal mismatch in data sources, where grandchild care data were drawn from the CHARLS 2018, while other key variables were obtained from the CHARLS 2020 may have contributed to this result. While grandchild caregiving in China is generally stable over time, variations in caregiving intensity and involvement may not have been fully captured. Future studies should employ fully longitudinal datasets or time-series analyses to better understand the evolving impact of grandchild care on cognitive function.

## Data Availability

The original contributions presented in the study are included in the article/supplementary material, further inquiries can be directed to the corresponding authors.

## References

[B1] Barragán A. Lucumí D. Lawlor B. (2021). Association of leisure activities with cognitive impairment and dementia in older adults in Colombia: A SABE-based study. *Front. Neurol.* 12:629251. 10.3389/fneur.2021.629251 33732207 PMC7956952

[B2] Bourassa K. Memel M. Woolverton C. Sbarra D. (2017). Social participation predicts cognitive functioning in aging adults over time: Comparisons with physical health, depression, and physical activity. *Aging Ment. Health* 21 133–146. 10.1080/13607863.2015.1081152 26327492

[B3] Brown C. Gibbons L. Kennison R. Robitaille A. Lindwall M. Mitchell M. (2012). Social activity and cognitive functioning over time: A coordinated analysis of four longitudinal studies. *J. Aging Res.* 2012 287438. 10.1155/2012/287438 22991665 PMC3444000

[B4] Chen C. Tian Y. Ni L. Xu Q. Hu Y. Peng B. (2024). The influence of social participation and depressive symptoms on cognition among middle-aged and older adults. *Heliyon* 10:e24110. 10.1016/j.heliyon.2024.e24110 38293386 PMC10825423

[B5] Chopik W. (2016). The benefits of social technology use among older adults are mediated by reduced loneliness. *Cyberpsychol. Behav. Soc. Netw.* 19 551–556. 10.1089/cyber.2016.0151 27541746 PMC5312603

[B6] Coall D. Hertwig R. (2010). Grandparental investment: Past, present, and future. *Behav. Brain Sci.* 33 1–19. 10.1017/S0140525x09991105 20377929

[B7] Di Tella S. Cabinio M. Isernia S. Blasi V. Rossetto F. Saibene F. (2021). Neuroimaging biomarkers predicting the efficacy of multimodal rehabilitative intervention in the Alzheimer’s dementia continuum pathology. *Front. Aging Neurosci.* 13:735508. 10.3389/fnagi.2021.735508 34880742 PMC8645692

[B8] Du Y. Luo Y. Zheng X. Liu J. (2023). Number of children and cognitive function among Chinese menopausal women: The mediating role of depressive symptoms and social participation. *J. Affect. Disord.* 340 758–765. 10.1016/j.jad.2023.08.084 37591349

[B9] Everly J. Plummer J. Lohman M. Neils-Strunjas J. A. (2023). Tutorial for speech-language pathologists: Physical activity and social engagement to prevent or slow cognitive decline in older adults. *Am. J. Speech Lang. Pathol.* 32 83–95. 10.1044/2022_Ajslp-22-00035 36450149

[B10] Fabbri L. Mosca I. Gerli F. Martini L. Pancani S. Lucidi G. (2018). The Games for older adults active life (GOAL) project for people with mild cognitive impairment and vascular cognitive impairment: A study protocol for a randomized controlled trial. *Front. Neurol.* 9:1040. 10.3389/fneur.2018.01040 30687208 PMC6336896

[B11] Fang E. Scheibye-Knudsen M. Jahn H. Li J. Ling L. Guo H. (2015). A research agenda for aging in China in the 21st century. *Ageing Res. Rev.* 24 197–205. 10.1016/j.arr.2015.08.003 26304837 PMC5179143

[B12] Fang E. Xie C. Schenkel J. Wu C. Long Q. Cui H. (2020). A research agenda for ageing in China in the 21st century (2nd edition): Focusing on basic and translational research, long-term care, policy and social networks. *Ageing Res. Rev.* 64:101174. 10.1016/j.arr.2020.101174 32971255 PMC7505078

[B13] Fu C. Li Z. Mao Z. (2018). Association between social activities and cognitive function among the elderly in China: A cross-sectional study. *Int. J. Environ. Res. Public Health* 15:231. 10.3390/ijerph15020231 29385773 PMC5858300

[B14] Gao Y. Jia Z. Zhao L. Han S. (2023). The effect of activity participation in middle-aged and older people on the trajectory of depression in later life: National cohort study. *JMIR Public Health Surveill.* 9:e44682. 10.2196/44682 36951932 PMC10131905

[B15] George K. Lutsey P. Kucharska-Newton A. Palta P. Heiss G. Osypuk T. (2020). Life-course individual and neighborhood socioeconomic status and risk of dementia in the atherosclerosis risk in communities neurocognitive study. *Am. J. Epidemiol.* 189 1134–1142. 10.1093/aje/kwaa072 32383452 PMC7666419

[B16] Gu S. Du X. Han D. Li S. Zhao J. Wu Y. (2024). The mediating roles of depressive symptoms and social participation in the relationship between the effects of pain and cognitive function among Chinese older adults: A longitudinal study. *Geriatric Nursing.* 57 147–153. 10.1016/j.gerinurse.2024.04.006 38657396

[B17] Harling G. Kobayashi L. Farrell M. Wagner R. Tollman S. Berkman L. (2020). Social contact, social support, and cognitive health in a population-based study of middle-aged and older men and women in rural South Africa. *Soc. Sci. Med.* 260:113167. 10.1016/j.socscimed.2020.113167 32688161 PMC7441312

[B18] Hendriks S. Peetoom K. Bakker C. van der Flier W. Papma J. Koopmans R. (2021). Global prevalence of young-onset dementia: A systematic review and meta-analysis. *JAMA Neurol.* 78 1080–1090. 10.1001/jamaneurol.2021.2161 34279544 PMC8290331

[B19] Hou X. Xiong Y. Qiao G. Zhou J. (2023). Association between caring for grandchildren based on living arrangements and cognitive function among Chinese middle-aged and older adults: The mediating roles of social activities and depressive symptoms. *Front. Public Health* 11:1105066. 10.3389/fpubh.2023.1105066 36866086 PMC9971921

[B20] Hughes M. Waite L. LaPierre T. Luo Y. (2007). All in the family: The impact of caring for grandchildren on grandparents’ health. *J. Gerontol. Ser. B Psychol. Sci. Soc. Sci.* 62 S108–S119. 10.1093/geronb/62.2.S108 17379680 PMC2562755

[B21] Kang S. Lim J. Park H. (2018). Relationship between low handgrip strength and quality of life in Korean men and women. *Soc. Sci. Med.* 27 2571–2580. 10.1007/s11136-018-1920-6 29922911

[B22] Kelly M. Duff H. Kelly S. Power J. Brennan S. Lawlor B. (2017). The impact of social activities, social networks, social support and social relationships on the cognitive functioning of healthy older adults: A systematic review. *Syst. Rev.* 6:259. 10.1186/s13643-017-0632-2 29258596 PMC5735742

[B23] Kim Y. Lee S. (2019). Social network types and cognitive decline among older Korean adults: A longitudinal population-based study. *International J. Geriatric Psychiatry* 34 1845–1854. 10.1002/gps.5200 31418470

[B24] Koczwara A. Patterson F. Zibarras L. Kerrin M. Irish B. Wilkinson M. (2012). Evaluating cognitive ability, knowledge tests and situational judgement tests for postgraduate selection. *Med. Educ.* 46 399–408. 10.1111/j.1365-2923.2011.04195.x 22429176

[B25] Kremen W. Elman J. Panizzon M. Eglit G. Sanderson-Cimino M. Williams M. (2022). Cognitive reserve and related constructs: A unified framework across cognitive and brain dimensions of aging. *Front. Aging Neurosci.* 14:834765. 10.3389/fnagi.2022.834765 35711905 PMC9196190

[B26] Lam B. Haslam C. Steffens N. Yang J. Haslam S. Cruwys T. (2020). Longitudinal evidence for the effects of social group engagement on the cognitive and mental health of chinese retirees. *J. Gerontol. Ser. B Psychol. Sci. Soc. Sci.* 75 2142–2151. 10.1093/geronb/gbz134 31630187

[B27] Li Y. Bai X. Chen H. (2022). Social isolation, cognitive function, and depression among chinese older adults: Examining internet use as a predictor and a moderator. *Front. Public Health* 10:809713. 10.3389/fpubh.2022.809713 35359786 PMC8963936

[B28] Liao S. Qi L. Xiong J. Yan J. Wang R. (2021). Intergenerational ties in context: Association between caring for grandchildren and cognitive function in middle-aged and older Chinese. *Int. J. Environ. Res. Public Health.* 18:21. 10.3390/ijerph18010021 33375149 PMC7792947

[B29] Liu Y. Liu Z. Liang R. Luo Y. (2022). The association between community-level socioeconomic status and cognitive function among Chinese middle-aged and older adults: A study based on the China Health and Retirement Longitudinal Study (CHARLS). *BMC Geriatr.* 22:239. 10.1186/s12877-022-02946-3 35317733 PMC8941774

[B30] Lv X. Li W. Ma Y. Chen H. Zeng Y. Yu X. (2019). Cognitive decline and mortality among community-dwelling Chinese older people. *BMC Med.* 17:63. 10.1186/s12916-019-1295-8 30871536 PMC6419492

[B31] Ma X. Piao X. Oshio T. (2020). Impact of social participation on health among middle-aged and elderly adults: Evidence from longitudinal survey data in China. *BMC Public Health* 20:502. 10.1186/s12889-020-08650-4 32295560 PMC7161098

[B32] Mao C. Li Z. Lv Y. Gao X. Kraus V. Zhou J. (2020). Specific leisure activities and cognitive functions among the oldest-old: The Chinese longitudinal healthy longevity survey. *J. Gerontol. Biol.* 75 739–746. 10.1093/gerona/glz086 30946444 PMC6776703

[B33] Posis A. Shadyab A. Parada H. Alcaraz J. Kremen W. McEvoy L. (2024). Multimorbidity, social engagement, and age-related cognitive decline in older adults from the rancho bernardo study of healthy aging. *J. Alzheimers Dis.* 97 1689–1702. 10.3233/Jad-230809 38306034 PMC10922723

[B34] Rafael A. Sousa L. Martins S. Fernandes L. (2021). Cognitive impairment in grandparents: A systematic review. *Psychiatry Investig.* 18 593–602. 10.30773/pi.2021.0034 34340272 PMC8328831

[B35] Richards M. James S. Sizer A. Sharma N. Rawle M. Davis D. (2019). Identifying the lifetime cognitive and socioeconomic antecedents of cognitive state: Seven decades of follow-up in a British birth cohort study. *Bmj Open* 9:e024404. 10.1136/bmjopen-2018-024404 31023749 PMC6502022

[B36] Samuel P. Marsack C. Johnson L. LeRoy B. Lysack C. Lichtenberg P. (2017). Impact of grandchild caregiving on african American grandparents. *Occup. Ther. Health Care* 31 1–19. 10.1080/07380577.2016.1243821 27805833 PMC5290223

[B37] Shah H. Albanese E. Duggan C. Rudan I. Langa K. Carrillo M. (2016). Research priorities to reduce the global burden of dementia by 2025. *Lancet Neurol.* 15 1285–1294. 10.1016/S1474-4422(16)30235-6 27751558

[B38] Sheng K. Chen H. Qu X. (2024). The effects of cognitive leisure activities on frailty transitions in older adults in China: A CHARLS-based longitudinal study. *BMC Public Health* 24:1405. 10.1186/s12889-024-18889-w 38802740 PMC11129477

[B39] Shi L. Tao L. Chen N. Liang H. (2023). Relationship between socioeconomic status and cognitive ability among Chinese older adults: The moderating role of social support. *Int. J Equity Health* 22:70. 10.1186/s12939-023-01887-6 37095501 PMC10124054

[B40] Shu C. Zheng C. Du X. Luo D. (2024). Exploring the role of vitamin D in cognitive function: Mediation by depression with diabetes modulation in older US adults, a NHANES weighted analysis. *Front. Nutr.* 11:1356071. 10.3389/fnut.2024.1356071 38895660 PMC11183290

[B41] Shu J. Qiang Q. Yan Y. Wen Y. Ren Y. Wei W. (2021). Distinct patterns of brain atrophy associated with mild behavioral impairment in cognitively normal elderly adults. *Int. J. Med. Sci.* 18 2950–2956. 10.7150/ijms.60810 34220322 PMC8241773

[B42] Smith L. Shin J. Sanchez G. Oh H. Kostev K. Jacob L. (2022). Social participation and mild cognitive impairment in low- and middle-income countries. *Preventive Med.* 164:107230. 10.1016/j.ypmed.2022.107230 36057392

[B43] Su X. Huang X. Jin Y. Wan S. Han Z. (2018). The relationship of individual social activity and cognitive function of community Chinese elderly: A cross-sectional study. *Neuropsychiatric Dis. Treatment* 14 2149–2157. 10.2147/Ndt.S160036 30197518 PMC6113942

[B44] Tarraf W. Kaplan R. Daviglus M. Gallo L. Schneiderman N. Penedof F. (2020). Cardiovascular risk and cognitive function in middle-aged and older hispanics/latinos: Results from the hispanic community health study/study of latinos (HCHS/SOL). *J. Alzheimers Dis.* 73 103–116. 10.3233/Jad-190830 31771064 PMC7412739

[B45] Van Bogart K. Harrington E. Witzel D. Kang J. Sliwinski M. Engeland C. (2024). Momentary loneliness and intrusive thoughts among older adults: The interactive roles of mild cognitive impairment and marital status. *Aging Ment Health* 28 1785–1792. 10.1080/13607863.2024.2368643 38907581 PMC11560736

[B46] Wang G. (2023). Association of hearing impairment and cognitive impairment with all-cause mortality among the oldest-old Chinese people. *Minerva Medica. Ear Hear.* 44 1212–1220. 10.23736/S0026-4806.23.08707-4 37382515

[B47] Wang J. Li S. Hu Y. Ren L. Jiang Y. Yu M. (2024). The bi-directional relationships between diversified leisure activity participation and cognitive function in older adults in China: Separating between-person effects from within-person effects. *BMC Geriatr.* 24:426. 10.1186/s12877-024-04997-0 38741042 PMC11092250

[B48] Wang M. Sung H. Liu J. (2022). Population aging and its impact on human wellbeing in China. *Front. Public Health* 10:883566. 10.3389/fpubh.2022.883566 35419339 PMC8995787

[B49] Wang S. Li S. Hu W. (2022). Grandparenting and subjective well-being in China: The moderating effects of residential location, gender, age, and income. *Soc. Sci. Med.* 315:115528. 10.1016/j.socscimed.2022.115528 36399982

[B50] Wang S. Lin J. Kuang L. Yang X. Yu B. Cui Y. (2024). Risk factors for social isolation in older adults: A systematic review and meta-analysis. *Public Health Nurs.* 41 200–208. 10.1111/phn.13266 38037451

[B51] Wang Y. Dou L. Wang N. Zhao Y. Nie Y. (2024). An analysis of factors influencing cognitive dysfunction among older adults in Northwest China based on logistic regression and decision tree modelling. *BMC Geriatr.* 24:405. 10.1186/s12877-024-05024-y 38714934 PMC11077840

[B52] Wilson R. Scherr P. Schneider J. Tang Y. Bennett D. (2007). Relation of cognitive activity to risk of developing Alzheimer disease. *Neurology* 69 1911–1920. 10.1212/01.wnl.0000271087.67782.cb 17596582

[B53] Xu J. Wang J. Wimo A. Fratiglioni L. Qiu C. (2017). The economic burden of dementia in China, 1990-2030: Implications for health policy. *Bull World Health Organ.* 95 18–26. 10.2471/BLT.15.167726 28053361 PMC5180346

[B54] Xu X. Xu Y. Shi R. (2024). Association between obesity, physical activity, and cognitive decline in Chinese middle and old-aged adults: A mediation analysis. *BMC Geriatr.* 24:54. 10.1186/s12877-024-04664-4 38212676 PMC10785530

[B55] Yang H. Zhang S. Zhang S. Wu Y. Luo R. (2022). Fertility experiences and later-life cognitive function among older adults in China. *Am. J. Hum. Biol.* 34:e23786. 10.1002/ajhb.23786 35929732

[B56] Zeng Y. Chen Y. Lum T. (2021). Longitudinal impacts of grandparent caregiving on cognitive, mental, and physical health in China. *Aging Ment. Health* 25 2053–2060. 10.1080/13607863.2020.1856779 33291945

[B57] Zhang R. Shen L. Miles T. Shen Y. Cordero J. Qi Y. (2020). Association of low to moderate alcohol drinking with cognitive functions from middle to older derAge among US adults. *JAMA Netw. Open* 3:e207922. 10.1001/jamanetworkopen.2020.7922 32597992 PMC7324954

[B58] Zhao Y. Hu Y. Smith J. Strauss J. Yang G. (2014). Cohort profile: The China health and retirement longitudinal study (CHARLS). *Int. J. Epidemiol.* 43 61–68. 10.1093/ije/dys203 23243115 PMC3937970

[B59] Zhu D. Al Mahmud A. Liu W. (2023). Social connections and participation among people with mild cognitive impairment: Barriers and recommendations. *Front. Psychiatry* 14:1188887. 10.3389/fpsyt.2023.1188887 37476544 PMC10356108

